# Possible changes in the transmissibility of trachoma following MDA and transmission reduction: implications for the GET2020 goals

**DOI:** 10.1186/s13071-015-1133-6

**Published:** 2015-10-22

**Authors:** Manoj Gambhir, Amy Pinsent

**Affiliations:** Department of Epidemiology and Preventive Medicine, Monash University, Melbourne, Australia

**Keywords:** Trachoma, GET2020, Immunity, Elimination, Reproduction number

## Abstract

**Background:**

The role of mass drug administration (MDA) and the implementation of transmission reduction measures are essential to successfully control and eliminate a wide range of NTDs, including the ocular disease trachoma. Immunity to trachoma infection acts by reducing the duration of an individual’s infectious period and by reducing their infectivity with each successive infection.

**Methods:**

In this study, we use a model of trachoma infection, which includes population immunity, to explore the impact of treatment and transmission reduction measures on trachoma prevalence. Specifically, we investigate the possibility of increasing transmissibility of trachoma arising as MDA and transmission reduction measures are scaled up in endemic settings.

**Results:**

We demonstrate this increase in transmissibility by calculating the effective reproduction number during several simulated control programmes and show that it is related to a decrease in the level of immunity in the population.

**Conclusions:**

This effect should be studied in the field by measuring the rate of return of infection and disease in at least two separate age groups. If the decline of population immunity is operating, it should be accounted for when planning for the GET2020 goal of eliminating blinding trachoma by 2020.

**Electronic supplementary material:**

The online version of this article (doi:10.1186/s13071-015-1133-6) contains supplementary material, which is available to authorized users.

## Background

Infection with ocular *Chlamydia trachomatis* (trachoma) remains the world’s leading infectious cause of blindness. It is endemic in 53 countries, with an estimated 84 million visually impaired and in need of treatment, while 1.2 million individuals are irreversibly blind [1]. The World Health Organization (WHO) recommends the use of the SAFE strategy to help with trachoma control (Surgery, Antibiotics, Facial cleanliness, and Environmental changes). Furthermore, it is estimated that 110 million people inhabit areas in which trachoma is believed to be endemic, and where the SAFE strategy needs to be implemented [2]. The WHO advocates eliminating blinding trachoma by 2020, and the Global Alliance for the Elimination of Trachoma by 2020 was established to help achieve this goal. The first GET2020 goal aims to reduce trachomatous inflammation follicular (TF) prevalence to <5 % in all 1–9 year olds by 2020. Reduced prevalence of active disease will only be maintained if transmission is reduced sufficiently, and this relies on the successful implementation of antibiotic treatment as well as long term reductions in overall transmission.

The role of mass drug administration (MDA) and the implementation of transmission reduction measures are essential to successfully control and eliminate a wide range of infectious diseases, such as: malaria [3], trachoma [4], schistosomiasis [5–7], onchocerciasis [8], and lymphatic filariasis [9]. However, for diseases in which immunity to infection is acquired through repeated infections, it has been suggested that dramatically lowering transmission in a short time period may lead to unintended outcomes on the prevalence of infection and disease [3, 10–13]; if transmission is reduced, individuals who have not recently experienced an infection may have less immune protection than before transmission was reduced [6, 7, 12].

Immunity to trachoma acts on at least two levels: first, by reducing an individual’s bacterial load and therefore infectivity [14–16], and second, by reducing the duration of an individual’s infectious period with successive infections [17, 18]. As such, if the rate at which new infections are acquired is reduced, the rate at which immunity to infection develops will also be reduced. As an individual’s infectivity and duration of infectiousness are key components of the basic reproduction number (*R*_0_), alterations at the population level of these parameters may impact the value of the effective reproduction number over time.

The possible adverse impact of MDA on *R*_0_ and short term immunity to trachoma was explored in an analysis by Liu et al. [19]. In this study a stochastic mathematical model was fitted to prevalence data from an MDA trial conducted across 32 communities in Tanzania [20, 21]. No significant change in the value of *R*_0_ across the 3 year trial period was identified, suggesting little to no loss of population immunity during this time [19]. However, if loss of population immunity were playing a role, a clear signal of increasing *R*_0_ may only become apparent after several years of treatment, and the initial increase may be small. Data from a single community does not therefore provide sufficient evidence to suggest that this outcome is not a possibility in other communities.

The effect of reduced population immunity as a consequence of intensive treatment has also been hypothesised for genital *C. trachomatis* infection. Brunham et al. [22, 23] observed that following the introduction of an intensive treatment programme in Greater Vancouver, British Colombia, paradoxically, a monotonic decrease in genital chlamydial prevalence over time was not observed. Rather, they observed annual increases in rates of re-infection, with 14 % of all cases reported annually being re-infections [22, 23]. They postulated that this observation in part arose as a consequence of early treatment interfering with the development of natural immunity to infection. This phenomenon has been named *The Arrested Immunity Hyposthesis* [23], since it hinges upon the successful clearance of infection at the individual level, but the unintended prevention (or ‘arrest’) of the development of population immunity.

In this study, we use a published model of trachoma infection, which includes the development of population immunity, to explore the impact of treatment and transmission reduction measures on trachoma prevalence. We also investigate the possibility of adverse outcomes that may arise as MDA and transmission reduction measures are scaled up in endemic settings in order to achieve the GET2020 goal of eliminating blinding trachoma. This modelling study is complemented by a second study on trachoma in this issue, which focuses on the utility of active disease prevalence for the purposes of forecasting trachoma [24].

## Methods

### Model and assumptions

**Fig. 1:**
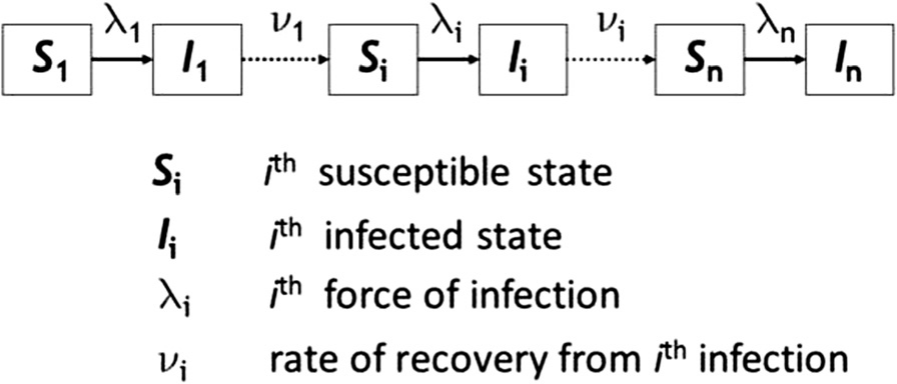
Schematic diagram of the compartmental model of trachoma epidemiology analysed here: the ladder of infection model

The model analysed here represents ocular (re) infection of individuals with *C. trachomatis* as they come into contact with other infected individuals; it uses a modified compartmental susceptible-infected-susceptible (*S-I-S*) structure, with the second *S* state being distinct from the original *S*, thereby constituting a ‘ladder of infection’ whose rungs represent successive infections, *i* (Fig. 1, and Additional file 1). The rate of recovery from an episode of infection increases with each successive infection (hence the duration of infection decreases following each successive infection) [17]. The model incorporates this effect by assuming that with each successive infection the acquired immune response to infection is enhanced [25], resulting in increasingly rapid clearance of bacteria. Additionally, we assumed that the infectivity of individuals declines with each successive infection and this effect is also likely to be a consequence of developed acquired immunity [14, 15]. Infectivity is assumed to be proportional to the bacterial infection load an individual carries. Both of these acquired immunity-modulated effects are modelled as exponential functions of the number of successive infections, *i*, experienced by an individual [26] (Fig. 1). All parameter values used are provided in the Additional file 1: Table S1. The original study [26] that outlined this model simultaneously fitted the parameters of these immunological functions, and an overall transmission parameter *β*, to age-dependent infection prevalence, duration of infection, and bacterial load data from a set of hyperendemic communities in Tanzania and Gambia. The model exhibited disease sequelae incidence rates that closely matched those published for Kongwa district, Tanzania, prior to the commencement of an intensive intervention study [27].

### The effect of MDA on the prevalence of infection

We applied two different simulated MDA regimens to the modelled population; 3 and 10 annual rounds. Each MDA round had a population coverage level, *c*, of 85 % and antibiotic efficacy, *e*, of 95 % as in the study by Gambhir et al. [28]. Those individuals in the population for whom an MDA round was successful cleared their infection completely and this was simulated here, as in previous studies, by shifting the cleared group (a proportion of the population *c* × *e*) from infected compartment *i* into susceptible compartment *i* on the ladder of infection.

### Modelling reductions in transmission

The third GET2020 goal states that the F & E components of the SAFE strategy should be enhanced in order to help achieve the elimination of blinding trachoma by 2020. In this study we model enhancement of the F & E components as an instantaneous drop in the transmission parameter *β* when MDA is initiated. However, it remains challenging for trachoma treatment trials to identify and demonstrate the efficacy of F & E interventions; therefore, exactly how much enhanced F & E interventions may reduce transmission remains unknown. A recent meta-analysis suggested that the potential contributions of F & E to the reduced odds of trachoma infection could be very important [29]. As such, we explore two values of transmission reduction (25 % and 50 %, one feasible and one optimistic; see e.g. [29]). The null assumption, i.e. no F &E impact, is considered here as those simulations that exclude F & E.

### Distribution of the population by the number of infections experienced

One of the main drivers of a possible change in the transmission rate relates to the level of population immunity. Immunity to infection (but not re-infection) dictates the rate of recovery from an infection episode, and the bacterial load carried by an individual upon infection. Here we model these quantities as a function of the number of prior infections experienced, *i* (Fig. 1). We illustrate the changing distribution of the population according to the number of prior infections, following each of the simulated interventions we apply, by plotting a histogram of the proportion of individuals who have experienced *i* infections. We calculated this quantity for the susceptible population, since the resulting changes in the distribution are easier to see, though we confirmed that the overall population (i.e. susceptible + infected) shift is very similar.

### Calculating the effective reproduction number over time

The summary parameter encapsulating the important aspects of transmissibility in the early stages of an outbreak is the basic reproduction number (*R*_0_). However, *R*_0_ would be the appropriate parameter to compute if the population were entirely susceptible and naïve to infection i.e. if there were no long term acquired immunity to infection in the population. Here, however, we are interested in the true transmissibility of trachoma infection in the presence of the current state of the population and so the relevant summary parameter is the effective reproduction number (*R*_*e*_), which accounts for population immunity. Here, *R*_*e*_ is a function of the current distribution of the population according to the number of prior infections experienced. We use the Next Generation Matrix method [30] to calculate *R*_*e*_ for our ladder of infection model structure (see Additional file 1).

## Results

### The effect of MDA on the prevalence of infection

**Fig. 2:**
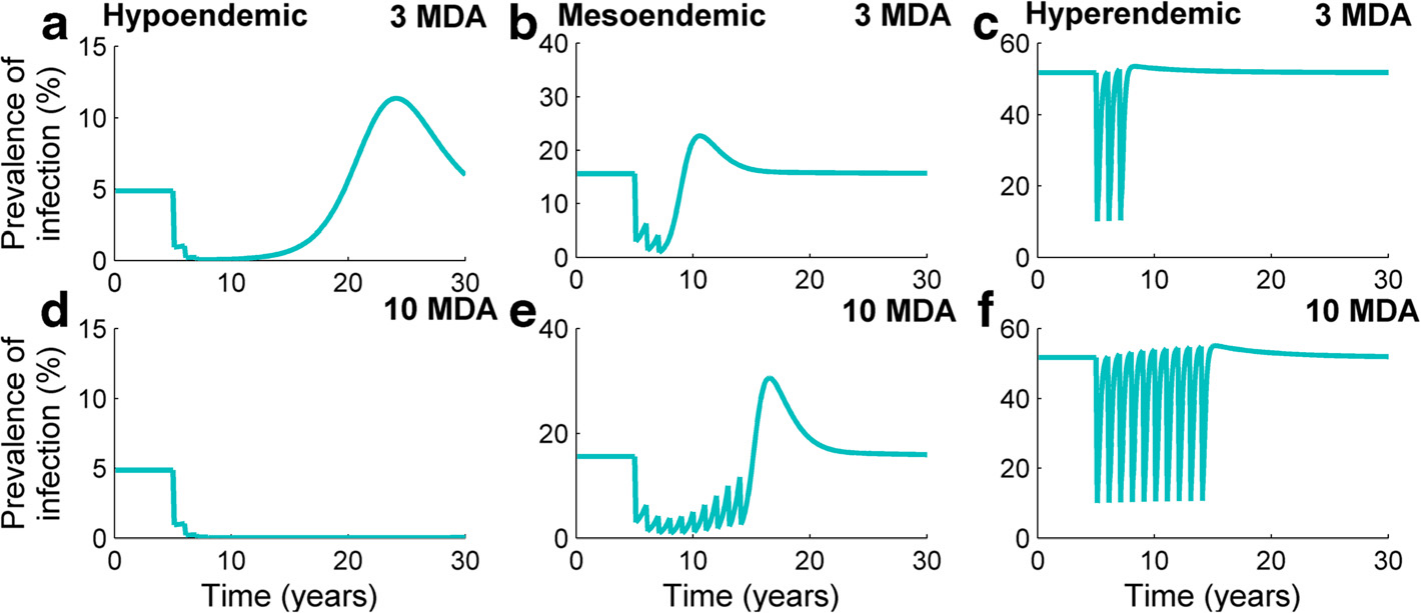
Annual MDA treatments in three endemic settings, impact and rebound Each endemic setting is subjected to 3 and 10 annual rounds of simulated MDA (with coverage and efficacy detailed in the main text, Methods). Infection is essentially eliminated following 3 and 10 rounds in the hypoendemic setting (**a** and **d**), but remains at a detectable level throughout the MDA programme in both meso (**b** and **e**)and hyperendemic (**c** and **f**) settings. The rate of rebound of infection increases over time in all settings but, once MDA ceases, its magnitude results in a large overshoot of infection prevalence beyond its baseline level in the hypo–and mesoendemic settings

We present six longitudinal time series following 3 (Fig. 2a-c) and 10 MDA rounds (Fig. 2d-f), the first at baseline, and the subsequent rounds at annual intervals. For each transmission setting we considered a prevalence of infection in the community at baseline to be approximately 50 %, 15 % and 5 % for hyper, meso and hypoendemic settings respectively (Fig. 2). Note that these baseline prevalence levels are slightly different to those used to fit the model published by Gambhir et al. [26] and were chosen to more clearly illustrate the results of the present study.

At the therapeutic coverage and drug efficacy levels chosen, infection prevalence rebounded within the course of one year following each MDA round in the hyperendemic setting (Fig. 2c and f). Stopping treatment after 10 consecutive annual rounds resulted in no appreciable overall decline in prevalence (Fig. 2f) as infection re-bounded to pre-intervention levels before the next round of MDA had begun. However, infection prevalence was reduced more dramatically in the mesoendemic setting (Fig. 2b and e), and infection rebound occurred more slowly between treatments for the first 3 rounds of MDA. Cessation of treatment 10 years after commencement also resulted in the re-emergence of infection (Fig. 2b and e). When 10 MDA rounds were given, in the hyper- and mesoendemic settings, the rate of rebound of infection became faster with each subsequent treatment round (Fig. 2e and f). However, this observed rate of increase was only apparent in the mesoendemic community after 3 rounds of MDA had already occurred. The model suggested that in the hypoendemic case, infection could be effectively eliminated after 3 annual treatment rounds. However, a rebound following cessation of treatment was seen here due to the continuation of transmission at very low prevalences (<<1 %) in our (deterministic) model. Note that a deterministic model does not lend itself to the exploration of the possibility that infection could fade out due to random chance, nor can we assess the possibility that infection in the community can resurge when transmission is very low; this would require a stochastic modelling approach.

### Distribution of the population by number of infections experienced

**Fig. 3:**
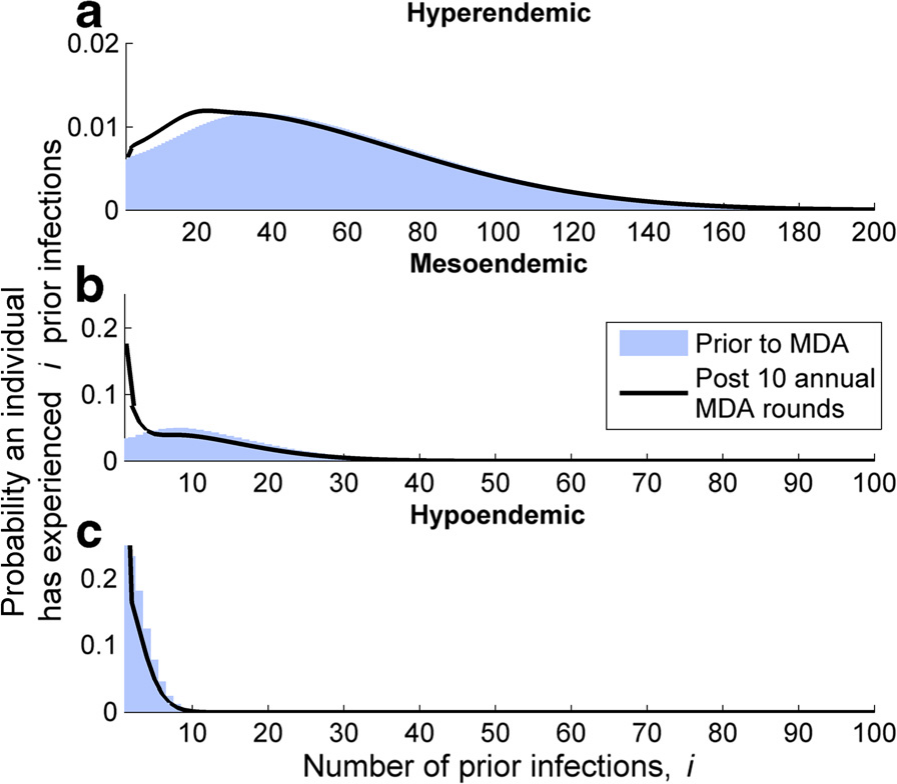
Distribution of the population according to the number of prior infections experienced. The susceptible population distribution is plotted for each endemic setting prior to (shaded distribution) and immediately following (black line) 10 MDA rounds. The effect of the MDA programme is to shift the population distribution to the left (i.e. to lower numbers of prior infections experienced), and this effect is more pronounced as the endemicity declines from hyper–to hypoendemic. Note the axis difference in **a** compared to **b** and **c**

The distribution of individuals along the ladder of infection was highly dependent upon the transmission parameter, *β*. As the value of *β* increased (high levels of *β* reflected higher transmission settings) the distribution of the number of infections experienced by individuals in the population became wider (Fig. 3). The maximum number of infections experienced by any individual in the hyperendemic setting is around 180, 40 in the mesoendemic, and 10 in the hypoendemic settings (Fig. 3). Despite a very high level of transmission, there remained a number of immunologically naive individuals who experienced very few previous infections in the hyperendemic community (Fig. 3a). These infection-naïve individuals would be expected to rapidly acquire infection and hence immunity, but as transmission is reduced, the rate at which this immunity is acquired is much slower.

The population distributions following 10 simulated MDA rounds (Fig. 3, dark blue lines) show a marked shift toward the zero end of the prior infection spectrum. This shift is greater for the lower endemic settings and results in an increase in the average recovery rate and infection load experienced by infected members of the population, hence the rate at which immunity is acquired is slower.

The biological effect of this population shift is illustrated in Table 1, where the average duration of infection and the infectivity, experienced by an average individual in the population, are both seen to increase immediately following MDA treatment. The increase in these average measures is greater following 10 annual MDA treatment rounds than it is for 3 rounds.

**Table 1:** Average duration of infection (in months) and infectivity (scaled between a high value of 1 and low of 0) calculated over the whole population at baseline and following 3 and 10 rounds of MDA, for each of the 3 endemic settings

	Hypoendemic		Mesoendemic		Hyperendemic	
	Duration	Infectivity	Duration	Infectivity	Duration	Infectivity
Initially	4.42	0.92	2.95	0.60	2.81	0.19
Post 3 MDAs	4.87	0.93	3.23	0.66	2.82	0.23
Post 10 MDAs	5.86	0.95	3.45	0.71	2.86	0.29

### Effective reproduction number over time

**Fig. 4:**
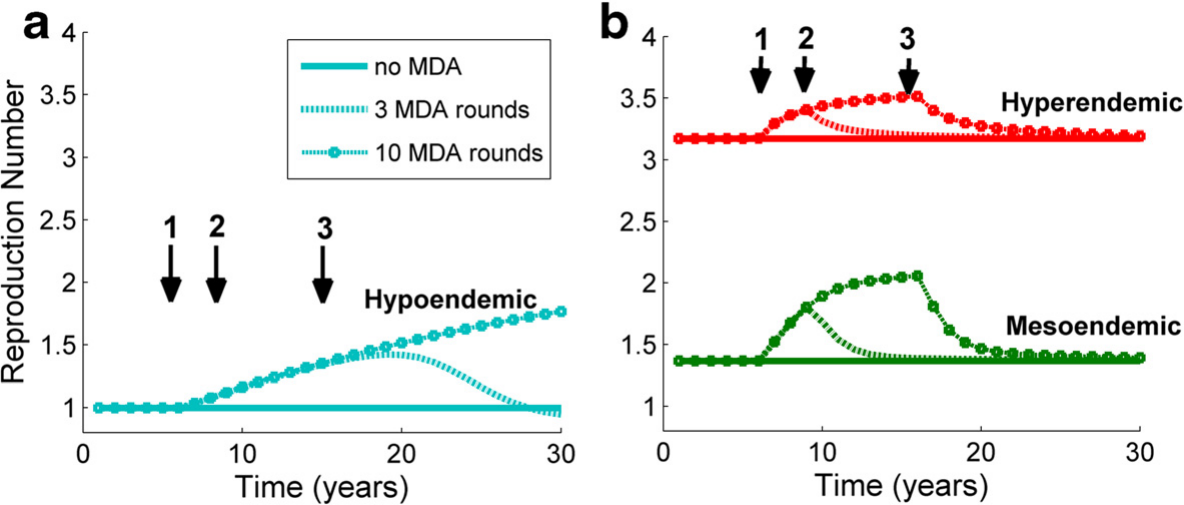
The time development of the *R*_*e*_ during and following MDA. The effective reproduction number *R*_*e*_ is plotted for time points prior to, during and following a 3 round and 10 round MDA programme for each endemic setting. The downward arrows indicate 3 important time points: 1) commencement of MDA, 2) cessation of 3 round MDA programme, 3) cessation of 10 round programme. In each setting there is a clear rise in the value of *R*_*e*_ over time after MDA begins, though this rise is more pronounced for lower endemic levels. The rise ends with the conclusion of MDA for the hyperendemic setting, but it persists beyond the conclusion of MDA as the endemic level declines. **a** indicates the change in *R*_*e*_ within the hypoendemic community. **b** in green the change in *R*_*e*_ within the mesoendemic community and in red the change in *R*_*e*_ within the hyperendemic community

The value of *R*_*e*_ rises steadily during the period when MDA is being applied and eventually falls back down to its baseline level (Fig. 4). The initial increase and then gradual decrease of *R*_*e*_ over time is larger when MDA is sustained for 10 rounds in comparison to 3. Additionally, the increase in *R*_*e*_ tends to continue beyond the duration of MDA as the endemicity decreases, with the extreme case shown in the upper curve of Fig. 4a (where 10 annual rounds of MDA are applied). Over 10 years of MDA within a hypoendemic community, a gradual increase in *R*_*e*_ from a baseline value of 1.0 to a final value of approximately 1.5 is observed, but this value continues to rise well after the cessation of MDA. This may mean that *R*_*e*_ is substantially elevated beyond its equilibrium level for substantially longer than the duration of treatment. Equally, even if local elimination of infection is achieved within the hypoendemic community, increases in population level *R*_*e*_ as transmission reduces may make these communities more susceptible to a large-scale outbreak if infection were to be re-introduced into the community.

### Transmission reduction and MDA together

**Fig. 5:**
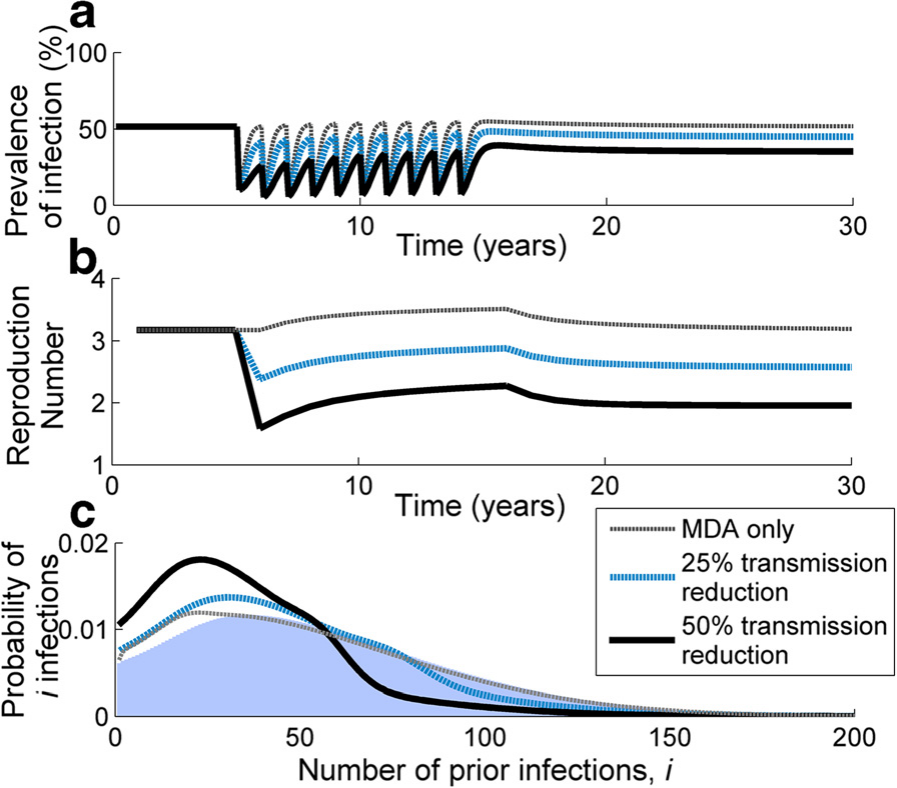
Impact and rebound of the prevalence of infection (**a**), the effective reproduction number (*R*_*e*_) (**b**), and the distribution of individuals according to their number of prior infections for 10 rounds of MDA (**c**). **(a)** The rate of rebound of infection increases over time but, once MDA ceases, infection initially overshoots then attains its new endemic equilibrium for both treatment regimens. **(b)** A similar time evolution of *R*_*e*_ is seen in both transmission reduction regimens: an abrupt drop with the transmission reduction, a gradual rise during MDA, followed by a fall and eventual settling to the new steady-state value. **(c)** The susceptible population distribution is plotted for the hyperendemic setting prior to (shaded distribution), immediately following (blue dashed line) 10 MDA rounds and 25 % transmission reduction, and immediately following (black solid line) 10 MDA rounds and 50 % transmission reduction. Note that the feint dotted grey line represents the ‘MDA only’ case with 10 treatment rounds

Exploring the impact of two simultaneous control interventions within a hyperendemic community—10 rounds of MDA and a transmission reduction of either 25 % or 50 % at baseline—we observed a steady increase in the rate of rebound of infection over time (Fig. 5a) following each round of MDA after transmission reduction was implemented; this was also reflected in the calculated value of *R*_*e*_ increasing over time (Fig. 5b). When comparing between the two different scenarios, the overall change in the population distribution per *i*^th^ infection was greater with transmission reduction and MDA (Fig. 5c) than seen when MDA was applied alone (Fig. 3a).

## Discussion

In this study we hypothesise that successive MDA treatment rounds and rapid transmission reduction measures may lead to an increase in transmissibility of trachoma infection, and an increase in difficulty in controlling disease. This could be due to the gradual loss of immunity from the population following treatment. This effect was most pronounced for the lowest prevalence communities, in which transmission was low and control may be achievable despite it. However, in these communities, while control is feasible, elimination may become markedly more difficult over time if *R*_*e*_ doubles over 20 years (as in Fig. 4). In hyperendemic communities, reductions in immunity result in a smaller transmissibility rise but, in the context of the extremely labour-intensive treatment schedules needed to achieve year-on-year declines in prevalence [14, 31, 32], even a marginal additional effort will be hard to maintain.

The increasingly rapid return of infection over time can be explained by the following process: first, sustained treatment prevents the population from progressing towards greater numbers of infections, which means that the population experiences fewer infections, on average; second, due to the slower rate of acquiring clearing immunity due to treatment and transmission reduction, individuals remain infected for longer periods of time, increasing the force of infection.

While our results in this study are theoretical, the rationale behind them has a sound biological basis. Recent work by Mitchell et al. [5] found that if protective immunity to schistosomiasis was short-lived, antibody levels declined to pre-intervention levels during or after MDA. However, if immunity was longer-lived and MDA was assumed to reduce transmission, a large over-shoot in measured egg count was observed. This modelling work was supported by antibody data suggesting MDA and transmission reduction may disrupt the development of protective immunity or alter existing population immunity [5].

The hypothesis presented in this article has been quantitatively tested just once for trachoma, to our knowledge, within a mesoendemic community, by Liu et al. [19]. While the authors did not identify a significant change in *R*_*e*_ over the 3 year period analysed, the analysis presented here suggests that a strong signal of increase in *R*_*e*_ may only become apparent after several years, and that its initial increase may be small.

Our simulated MDA assumes random allocation of doses in the population with each treatment, while a persistently untreated group of individuals is also a possibility. In the latter case, it is likely that the value of *R*_*e*_ would increase more slowly and to a lesser overall extent due to a more rapid reseeding of infection from the untreated group into the wider population following each treatment round.

The adverse effect we find applies to communities in which MDA is introduced in isolation, but it may be exacerbated when dramatic transmission reduction measures are rapidly implemented. Such an outcome has previously been suggested for malaria [10, 12]. In this instance it is expected that a higher number of severe disease cases will be observed immediately following the reduction in transmission, primarily as a result of the loss of population immunity. Furthermore, epidemiological surveillance data in genital chlamydia infection has also shown empirical evidence of this effect [22, 23]. It was reported that re-infection rates following the introduction of an infection control programme resulted in a 4.6 % per year re-infection rate increase during the period of study from 1989–2003 [22]. These findings were complemented with a mathematical transmission model suggesting that early treatment of infection increased the population’s susceptibility to re-infection [22]. However, Vickers et al. [33] suggested that the increased rates of re-infection in Canada could mainly be attributable to an increase in testing volume, implying that *The Arrested Immunity Hypothesis* had a less significant role on the transmission dynamics than previously reported [36].

To date, the analysis of serological data collected through trachoma surveillance has been very limited [34, 35]. Martin et al. [35] fitted a catalytic model to serological data in order to identify when changes in transmission intensity within a Tanzanian community occurred. The authors used a binary cut-off to determine whether individuals in the study were seropositive or seronegative and, during the surveillance period, they did not identify any individuals who seroreverted, suggesting no loss of population immunity. However, the use of a binary cut-off to define individuals as seropositive or seronegative can mask changes in the antibody titres that may occur over time, meaning that even if an individual’s titre has declined dramatically, if it does not fall below the cut-off threshold, they will still be classified as seropositive. Indeed, Goodhew et al. [34] showed that there was evidence of a significant decline in trachoma antigens for nearly all age groups 6 months after MDA was applied. Anti-trachoma antibodies are likely to be associated with protection although the exact relationship is poorly understood. Antibody titers may decrease over time while an individual remains seropositive leading to a likely decrease in protection over time.

Therefore, while the effects presented here remain to be substantiated with data from treatment and transmission reduction trachoma trials, we suggest the effect seen for genital chlamydia and schistosomiasis should be motivation to design trials which include these outcomes.

## Conclusions

### Implications for study design

In this article we have considered the impact of MDA and transmission reduction interventions across the whole community; however, the data analysed by Liu et al. in [19] were collected from 0–5 year olds. It is possible that, since young children experience the most ‘severe infections’ with respect to having the highest bacterial loads and slower rates of recovery, shifts in population level immunity may not manifest themselves in this age group. However, longer infection durations may be seen in older age groups which increasingly consist of individuals with less mature immunity as a result of declines in transmission; this older population was not observed in the analysis by Liu et al. [19]. Moreover, studies previously conducted to examine loss of immunity to *C. trachomatis*remain limited to the analysis of a small number of communities [22, 36], potentially limiting their power to identify loss of population immunity. This suggests that in order to monitor whether the GET2020 goals are being achieved, larger studies that also monitor the infection and disease status of older individuals in the population will be important to ensure that loss of immunity through transmission reduction does not help to sustain infection within the community.

It may be that a combination of transmission-enhancing and reducing (so called Allee [37, 38]) effects will determine the trajectory of the prevalence over time close to elimination [39]. Lietman et al. demonstrated that a common linear hazard of infection term in a mechanistic model is not sufficient to describe trachoma infection rebound dynamics at low prevalences, and they accounted for this using a nonlinear adjustment to the hazard [38]. It is incumbent upon modellers and quantitative parasitologists to help devise field studies to measure the rate of return of infection and disease in at least two separate age groups; these studies could determine whether density dependent effects are playing a role and what their relative contribution is.

### Implications for the GET2020 goals

The results we present apply directly to the GET2020 goals relating to the reduction of active disease (TF) and disease sequelae (TT): both MDA and transmission reduction (e.g. F & E) interventions may both result in rises in rates of return of infection and disease over time due to the loss of population immunity, making the achievement of the TF and TT goals more challenging.

In addition, we show that this effect may be worse for lower-endemic communities. However, we note that the model used in this analysis was deterministic, and therefore it is not ideally suited to assess the stochastic fade-out and elimination of infection within a community. Therefore the perverse outcome observed in lower transmission settings may not be a problem if infection fades out and does not eventually re-emerge in a community, as it does in our deterministic model. A reason that the observed effect is most exacerbated within the hypoendemic community is that we have assumed immunity to infection develops exponentially and, as such, larger changes in rates of recovery and infectivity will be seen when people within the community experience a low number of infections both at baseline and following MDA.

We also show the effect may also be worse for currently highly-endemic communities in which intensive SAFE programmes are imposed, as dictated by the third GET2020 goal, which states that the F & E components of the SAFE strategy should be enhanced. However, by reducing the frequency at which individuals become infected through transmission reduction and MDA, this will ultimately result in lower equilibrium levels of infection prevalence and ultimately a lower prevalence of disease sequelae due to individuals experiencing fewer infections over their lifetime.

The role of acquired immunity to trachoma remains poorly understood, but modelling the dynamics associated with the loss and gain of population immunity is essential if we are to account for outcomes that, perversely, may result in transmissibility increases as a result of transmission reduction measures. Changes in population immunity to infection as a result of intensive transmission reduction measures may frustrate the achievement of the GET2020 goals, and field trials need to be designed in order to monitor and minimise the chance and effects of these outcomes. This may also mean that longer-term post-validation surveillance is required to ensure the incidence of infection does not begin to increase over time, following achievement of the GET2020 goals. However, it is likely the cost of intermittent surveillance will be justified if we want to achieve the long-term elimination of blinding trachoma.

## Additional file

Additional file 1: **Supplementary Material.** (DOCX 256 kb)
