# Supplementary material for: Possible changes in the transmissibility of trachoma following MDA and transmission reduction: implications for the GET2020 goals
Source: Parasit Vectors. 2015 Oct 22;8:530. doi: 10.1186/s13071-015-1133-6 (PMC4618927; doi:10.1186/s13071-015-1133-6)
Supplement: Supplementary file 1 — Supplementary Material. (DOCX 256 kb) [file 13071_2015_1133_MOESM1_ESM.docx]

**Additional file 1: Supplementary Material**

***The model of trachoma transmission***

The model we use is made up from repeated susceptible and infected compartments, each given an index *i*. As individuals become infected and recover from infection, they move up a ‘ladder’ of infection through susceptible ($S_{i}$) and infected ($I_{i}$) compartments, each connected to the next compartment above. Discretised versions of the following continuous (in time and age) partial differential equations were used to describe the flow from one compartment to another in the computer simulation,

$\frac{\partial S_{i}(a,t)}{\partial t}+\frac{\partial S_{i}(a,t)}{\partial a}=-\beta S_{i}(a,t)\int_{a'} \frac{w(a,a')\sum_{j} \rho_{j}I_{j}(a',t)}{\sum_{j} I_{j}(a',t)+S_{j}(a',t)}da'-\mu(a)S_{i}(a,t)+\upsilon_{i-1}I_{i-1}(a,t)$ (1)

$\frac{\partial I_{i}(a,t)}{\partial t}+\frac{\partial I_{i}(a,t)}{\partial a}=\beta S_{i}(a,t)\int_{a'} \frac{w(a,a')\sum_{j} \rho_{j}I_{j}(a',t)}{\sum_{j} I_{j}(a',t)+S_{j}(a',t)}da'-\mu(a)I_{i}(a,t)-\upsilon_{i}I_{i}(a,t)$ (2)

$S_{i}$ and $I_{i}$ are functions of age *a* and time *t*;$\beta$ is the transmission parameter from infected to susceptible states and was estimated from fitting the model by maximum likelihood to the data; $w(a,a')$is a mixing matrix containing the information on the rate of mixing between individuals of age $a$ and $a'$ [[1](#_ENREF_1)],

$\left(w(a,a')=\varepsilon\delta_{a,a'}+(1-\varepsilon)\frac{N_{a'}}{\sum_{a'} N_{a'}}\right)$, (3)

$\delta_{a,a'}$ is the Kronecker Delta[[2](#_ENREF_2)]; $N_{a'}$ is the number of individuals of age $a'$ and $\varepsilon$ is a parameter that determines the population mixing patterns and it ranges from 0 (representing random mixing among age groups) to 1 (representing assortative mixing, i.e., each age group mixed only with itself); it is set to 0.5 in the model which represents an intermediate level of randomness and assortativity [[3](#_ENREF_3)]; $\rho_{j}$ is the infectivity of infected individuals in compartment $I_{j}$; $\mu(a)$ is the death-rate form individuals of age *a* and the death-rates used here corresponded with the 2001 WHO Tanzanian and Gambian life tables; and $\upsilon_{i}$ is the recovery rate from infection for those in compartment $I_{i}$.

*Recovery rate*

The per individual rate of recovery, denoted by$\upsilon_{i}$(measured as the rate per year), from infection *i*, is assumed to change as an exponential function of *i* that begins at a rate $\upsilon_{1}$ (for recovery from first infection) and rises to a maximum rate $\upsilon_{\infty}$ (for recovery from a large number of infections),

$\upsilon_{i}=\left(\upsilon_{1}-\upsilon_{\infty}\right) \exp\left[-\gamma\left(i-1\right) \right]+\upsilon_{\infty}$. (4)

The parameters of this exponential function, $\upsilon_{1}$, $\upsilon_{\infty}$, and $\gamma$ were estimated by fitting (by maximum likelihood estimation) the model to the mean duration of infection for different age groups using published data [[4](#_ENREF_4)] which have been recently re-analyzed [[5](#_ENREF_5)]. The results of these parameter estimations are shown in Table S1 ($\gamma$ is a rate constant that describes the rate of change of the recovery rate per infection).

*Infectivity*

We assume the infectivity of an individual, denoted by$\rho_{i}$, to be proportional to their bacterial load $l_{i}$, which we suggest is a function of the number of previous infections experienced by each individual in the population, a trend that is in agreement with the data from trachoma endemic communities in which the bacterial load decreases with age [[6-9](#_ENREF_6)]. To capture this decreasing load with the number of previous infections, we use an exponential function such that the load declines from its initial value $\begin{matrix} \\ l_{1} \end{matrix}$ as described below,

(5)

Parameter $\varphi$ can be estimated from age-stratified data on bacterial load by fitting equation (5) to the hyperendemic data (assumed to be at equilibrium) of West et al. [[8](#_ENREF_8)].

*Calculation of the Effective Reproduction Number R_e_*

We follow the approach outlined in the paper by Diekmann et al [[10](#_ENREF_10)] to calculate the basic reproduction number for the ladder of infection model detailed in this study. This method requires the construction of a Next Generation Matrix (NGM) for our model system (outlined in the 'Model Equations' section of the Appendix) and a calculation of the eigenvalues of this matrix. *R*_0_ is then equal to the largest eigenvalue, or spectral radius, of the matrix. We go about constructing the NGM by first considering the age-independent version of the model equations outlined above. By reducing the system to the infected states (i.e. the compartments labeled $I_{i}$), we have a system with *n* state variables consisting of the *n* infected states along the ladder of infection. We then populate a Transmission matrix ($\overline{T}$)—which includes all epidemiological ‘births’—and a Transition matrix ($\overline{E}$)—which contains all other flows between infected compartments.

In the model equations above, at the beginning of an epidemic (i.e. when the number of

infected individuals is very close to zero), each transmission term (i.e. epidemiological birth) contains

the transmission rate $\beta$, and the infectivity at a particular level of the ladder of infection$\rho_{i}$(we

ignore the mixing matrix for the moment). At any given moment in time, the susceptible

population may not be concentrated entirely in the *i*^th^ level of the ladder, so we also need to consider the fraction of the population that is situated on the *i*^th^ level. We denote this fraction $S_{i}^{0}$, where the ‘0’ superscript refers to the fact that we’re examining the situation at the zero time point i.e. at the beginning of the epidemic. Since each term contributing to the transmission terms for a particular level of the ladder, *i*, will need to be multiplied by the susceptible fraction available at that level, the transmission matrix $\overline{T}$ becomes:

$$\overline{T}=\beta.\left[\begin{matrix} S_{1}^{0}.\rho_{1} & S_{1}^{0}.\rho_{2} & \ldots& S_{1}^{0}.\rho_{i} & \ldots& S_{1}^{0}.\rho_{n} \\ S_{2}^{0}.\rho_{1} & S_{2}^{0}.\rho_{2} & \ldots& S_{2}^{0}.\rho_{i} & \ldots& S_{2}^{0}.\rho_{n} \\ \vdots& & \ddots& & & \vdots\\ S_{i}^{0}.\rho_{1} & S_{i}^{0}.\rho_{2} & \cdots& S_{i}^{0}.\rho_{i} & \cdots& S_{i}^{0}.\rho_{n} \\ \vdots& & & & \ddots& \vdots\\ S_{n}^{0}.\rho_{1} & S_{n}^{0}.\rho_{2} & \ldots& S_{n}^{0}.\rho_{i} & \ldots& S_{n}^{0}.\rho_{n} \end{matrix}\right]$$

For the complete age-structured model, we need to include the mixing matrix $w(a,a')$(hereafter denoted$\overline{w}$) that governs the relative rate of contact between individuals of age group $a$with those in age group$a'$. The system is *n* dimensional for the non age-structured model and—if there are *m* age groups, and each of these has to be accounted for at each prior infection level, *i*—we end up with an *n*×*m* dimensional system. Each of the elements in the $\overline{T}$matrix above is multiplied by the mixing matrix $\overline{w}$ to create an (*n*×*m)-*dimensional transmission matrix:

$$\overline{T}=\beta.\left[\begin{matrix} S_{1}^{0}.\rho_{1}.\overline{w} & S_{1}^{0}.\rho_{2}.\overline{w} & \ldots& S_{1}^{0}.\rho_{i}.\overline{w} & \ldots& S_{1}^{0}.\rho_{n}.\overline{w} \\ S_{2}^{0}.\rho_{1}.\overline{w} & S_{2}^{0}.\rho_{2}.\overline{w} & \ldots& S_{2}^{0}.\rho_{i}.\overline{w} & \ldots& S_{2}^{0}.\rho_{n}.\overline{w} \\ \vdots& & \ddots& & & \vdots\\ S_{i}^{0}.\rho_{1}.\overline{w} & S_{i}^{0}.\rho_{2}.\overline{w} & \cdots& S_{i}^{0}.\rho_{i}.\overline{w} & \cdots& S_{i}^{0}.\rho_{n}.\overline{w} \\ \vdots& & & & \ddots& \vdots\\ S_{n}^{0}.\rho_{1}.\overline{w} & S_{n}^{0}.\rho_{2}.\overline{w} & \ldots& S_{n}^{0}.\rho_{i}.\overline{w} & \ldots& S_{n}^{0}.\rho_{n}.\overline{w} \end{matrix}\right]$$

Aside from transitions corresponding to aging, demographic births and deaths (which we do not include in the algebra here for simplicity) the only transitions from the epidemiologically ‘active’ compartments are those which represent recovery from the infected state, and these are denoted by the parameter $\nu_{i}$.

For the simplified model, not including age stratification, our Transition matrix $\overline{E}$ is therefore given by the diagonal matrix:

$$\overline{E}=\left[\begin{matrix} \nu_{1} & 0 & \ldots& 0 & \ldots& 0 \\ 0 & \nu_{2} & \ldots& 0 & \ldots& 0 \\ \vdots& & \ddots& & & \vdots\\ 0 & 0 & \cdots& \nu_{i} & \cdots& 0 \\ \vdots& & & & \ddots& \vdots\\ 0 & 0 & \ldots& 0 & \ldots& \nu_{n} \end{matrix}\right]$$

And when we do include age stratification, the full (*n*×*m)* -dimensional transition matrix becomes:

$$\overline{E}=\left[\begin{matrix} \nu_{1}.\overline{I} & 0 & \ldots& 0 & \ldots& 0 \\ 0 & \nu_{2}.\overline{I} & \ldots& 0 & \ldots& 0 \\ \vdots& & \ddots& & & \vdots\\ 0 & 0 & \cdots& \nu_{i}.\overline{I} & \cdots& 0 \\ \vdots& & & & \ddots& \vdots\\ 0 & 0 & \ldots& 0 & \ldots& \nu_{n}.\overline{I} \end{matrix}\right]$$

Where $\overline{I}$is the *m*-dimensional identity matrix.

The NGM $\overline{K}$, is formulated as:

$$\overline{K}=\overline{T}.\overline{E}^{-1}$$

and the effective reproduction number is calculated as the largest eigenvalue of $\overline{K}$ .

*Model parameters*

Table S1 Parameter values used in the model, with 95% confidence intervals where appropriate.

| ***Parameter*** | ***Parameter definition*** | ***Maximum likelihood estimate ([95%CI] where appropriate)*** |
| --- | --- | --- |
| *Transmission dynamics parameters* | | |
| 1/$\upsilon_{1}$ | Mean duration of first infection | *15.1 [6.5,23.3] months* |
| 1**/**$\upsilon_{\infty}$ | Mean duration of infection after multiple prior infections | *2.8 [2.4, 3.2] months* |
| $\gamma$ | Rate of drop of duration of infection per prior infection | *0.7 [0.1,∞] infection^-1^* |
| $l_{1}$ | Infection load per person at first infection | *1.1 x 10^5^[0.9, 1.3 x 10^5^]copies omp1 per swab* |
| $\varphi$ | Rate of drop of infection load per prior infection | *0.05 [0.03, 0.07] infection^-1^* |
| $\beta$ | Transmission coefficient: the rate of transmission (per year) of infection between individuals | *27.7(hyper), 7.0 (meso), 3.5 (hypo) year^-1^* |
| *Treatment-related parameters* | | |
|  | Efficacy of single-dose azithromycin treatment | *95%* |
|  | *Population coverage of MDA* | *85%* |

***Sensitivity to immunity and infectivity parameters***

We tested the model’s sensitivity to two of the key parameters associated with immunity: the exponential decay rates of the recovery rate and infectivity, by setting these parameters to a high and low value. Our outcome measure was the effective reproduction number over time, which we compared with the *R*_e_ baseline time dependent profile. The model was subjected to 10 simulated MDA rounds, as in the main text, to determine the extent to which the observed phenomena were dependent upon particular parameter values. We found that the phenomenon of raised effective reproduction number was reproduced in each of the high and low parameter combinations, though the extent to which *R*_e_ was raised changed, and this change happened beginning from a different baseline, pre-treatment, *R*_e_ value.

The results show that decreasing the rate of decay of each of the parameters (top lines in Figure S1) raises the overall reproduction number value, but it also reduces the impact upon the baseline value of this parameter with treatment (note that the lines on the top figure are flatter than those on the bottom). This situation corresponds to a very slow development of acquired immunity upon increasing numbers of infections. Increasing the values of the decay constants so that the development of acquired immunity requires essentially one infection (bottom curves in Figure S1), results in an increased impact of treatment upon the reproduction number. The true development rate of acquired immunity is likely to lie in between these two extreme cases.


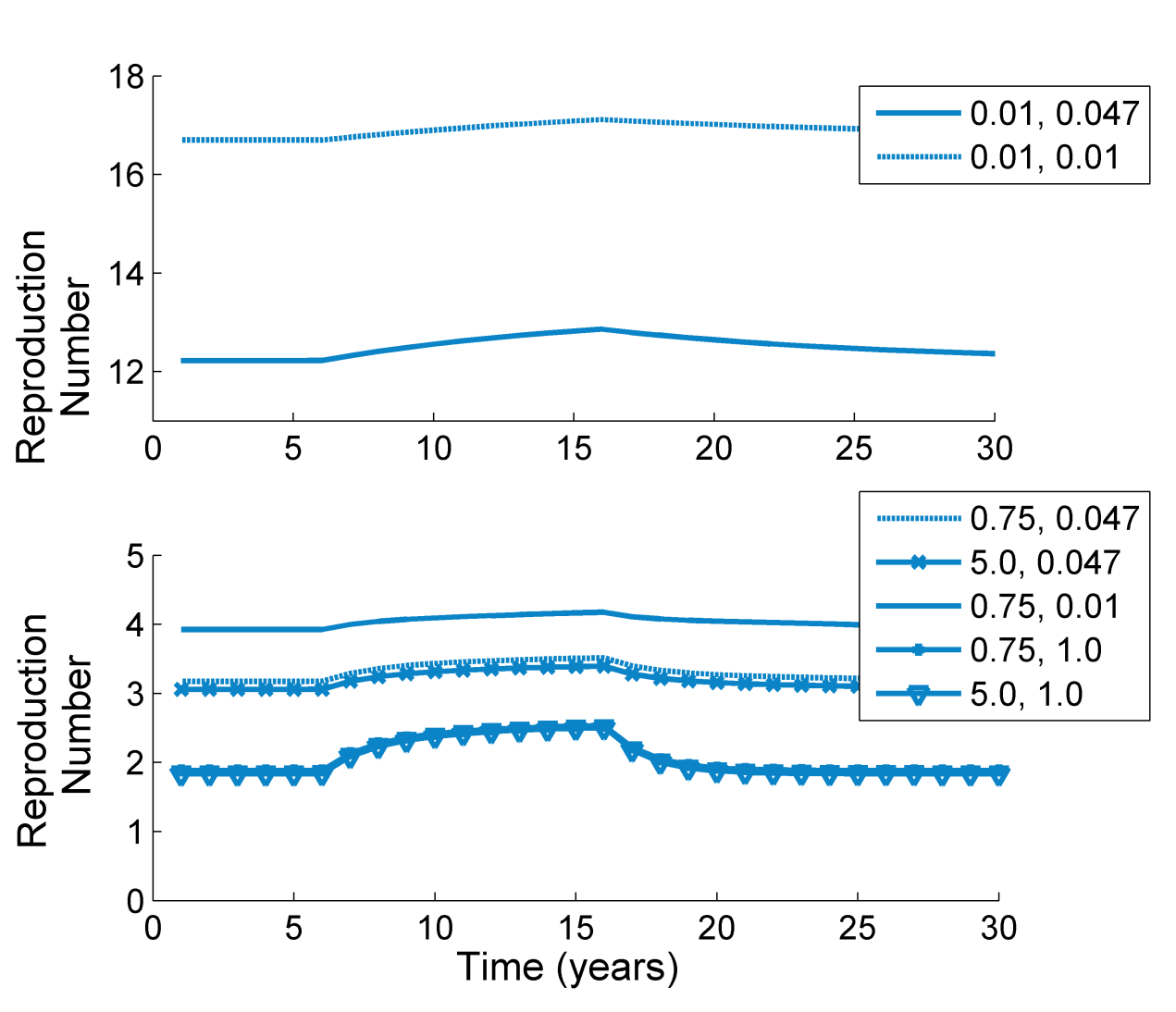


**Figure S1: Sensitivity plot of the change in the effective reproduction number over time before, during and following 10 simulated MDA rounds.** We varied the recovery rate and infectivity decay parameters as indicated in the figure legends (first number is the recovery rate decay, and the second number is the infectivity decay rate; the values used in the main text are: 0.75, 0.47, which correspond with the dotted line in the lower plot).

**References**

1. Anderson R, May R: **Infectious diseases of humans: dynamics and control**: Oxford University Press; 1992.

2. Boas ML: **Mathematical methods in the physical sciences**, 2nd edn. New York: John Wiley & Sons; 1983.

3. Gambhir M, Basanez MG, Turner F, Kumaresan J, Grassly NC: **Trachoma: transmission, infection, and control**. *The Lancet infectious diseases* 2007, **7**(6):420-427.

4. Bailey R, Duong T, Carpenter R, Whittle H, Mabey D: **The duration of human ocular *Chlamydia trachomatis* infection is age dependent**. *Epidemiol Infect* 1999, **123**(3):479-486.

5. Grassly NC, Ward ME, Ferris S, Mabey DC, Bailey RL: **The natural history of trachoma infection and disease in a Gambian cohort with frequent follow-up**. *PLoS Negl Trop Dis* 2008, **2**(12):e341.

6. Solomon AW, Holland MJ, Burton MJ, West SK, Alexander ND, Aguirre A, Massae PA, Mkocha H, Munoz B, Johnson GJ *et al*: **Strategies for control of trachoma: observational study with quantitative PCR**. *Lancet (London, England)* 2003, **362**(9379):198-204.

7. Solomon AW, Holland MJ, Alexander ND, Massae PA, Aguirre A, Natividad-Sancho A, Molina S, Safari S, Shao JF, Courtright P *et al*: **Mass treatment with single-dose azithromycin for trachoma**. *N Engl J Med* 2004, **351**(19):1962-1971.

8. West ES, Munoz B, Mkocha H, Holland MJ, Aguirre A, Solomon AW, Bailey R, Foster A, Mabey D, West SK: **Mass treatment and the effect on the load of *Chlamydia trachomatis* infection in a trachoma-hyperendemic community**. *Invest Ophthalmol Vis Sci* 2005, **46**(1):83-87.

9. Burton MJ, Holland MJ, Makalo P, Aryee EA, Alexander ND, Sillah A, Faal H, West SK, Foster A, Johnson GJ *et al*: **Re-emergence of *Chlamydia trachomatis* infection after mass antibiotic treatment of a trachoma-endemic Gambian community: a longitudinal study**. *Lancet (London, England)* 2005, **365**(9467):1321-1328.

10. Diekmann O, Heesterbeek JA, Roberts MG: **The construction of next-generation matrices for compartmental epidemic models**. *Journal of the Royal Society, Interface / the Royal Society* 2010, **7**(47):873-885.
